# Reducing Dependence on Non-Renewable Sources: The Addition of Binchotan Charcoal in Carbon Anodes for the Aluminum Industry

**DOI:** 10.1021/acsomega.5c02522

**Published:** 2025-06-13

**Authors:** Lucas C. Pereira, Cledson R. Corrêa, Eduardo O. Hias, Henrique C. Santos, Hiroyuki Yamamoto, João L. Barros, Fábio M. Yamaji

**Affiliations:** † 119519 Federal Institute of Education Science and Technology of São Paulo, Itapetininga 18202-000, Brazil; ‡ 613995 UFSCar − Campus Sorocaba, Sorocaba 18052-780, Brazil; § 311624 Brazilian Aluminum Company, Alumínio 18125-000, Brazil; ∥ 12965 Nagoya University, Nagoya 464-8601, Japan; ⊥ Federal Institute of Education Science and Technology of São Paulo, Sorocaba 18043-060, Brazil

## Abstract

Carbon anodes are produced by the mixture of calcined petroleum coke and coal tar pitch, forming a paste that is cooked lately. The addition of biomass in the anodes could help reduce greenhouse gas emissions and minimize the use of nonrenewable sources. In this work, carbon anodes were produced with the addition of 1 and 3% of binchotan charcoal, replacing part of the petroleum coke. Binchotan is a type of Japanese charcoal, characterized by having a high density, good electrical properties, and a high carbon content. The bioanodes were evaluated with the following analyses: apparent density, mechanical strength, CO_2_ reactivity, electrical resistivity, and chemical composition. The tests were performed based on a methodology that is used in the aluminum industry and the results were compared with a typical carbon anode and an international reference. All of the values were considered adequate, indicating that it is possible to replace small percentages of calcined petroleum coke with binchotan charcoal. Technically, binchotan charcoal seemed feasible to be applied in carbon anodes produced on a laboratory scale and has the potential to be used in the aluminum industry.

## Graphical abstract

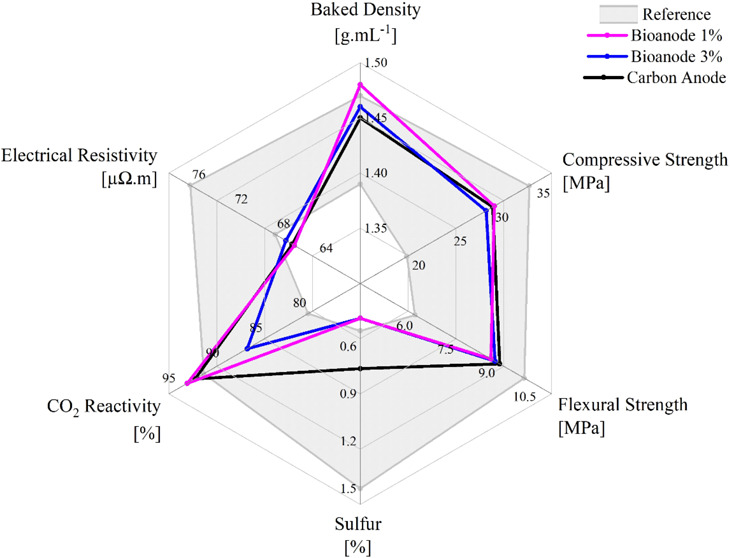

## 1 Introduction

Aluminum is the most abundant metal in the Earth’s crust, composing approximately 8% of the mass of the planet. It is rarely found in its metallic form due to a great affinity with oxygen, which gives origin to alumina. Among the lightweight metals and their alloys, aluminum is the most produced one, having a wide variety of applications and several properties.^1,2^

To produce 1 kg of aluminum, roughly 2 kg of alumina, 0.45 kg of carbon, and 14 kWh of electricity are consumed. In countries where the electrical matrix utilizes renewable sources, the environmental impacts can be reduced, and more sustainable efforts are focused on other processes, such as alumina refining and anode production.^3,4^

In general, the carbon anodes are produced from a mixture of calcined petroleum coke (≈70%) and coal tar pitch (≈30%), forming a paste that is molded in the desired shape and baked afterward. The coal tar pitch acts as a coke particle binder, forming an anode with good properties: high thermal and mechanical resistance, low electrical resistivity, and low reactivity.^5,6^ Calcination, made in petroleum coke, is a thermic process that aims to increase the fixed carbon content by reducing the contents of volatile matter and moisture, producing a material with high values of density, electrical conductivity, and mechanical strength.^7^

Studies related to the challenges of biomass use in the aluminum industry have been addressed in the literature. Biomass tends to be inferior to fossil materials and it is necessary to use treatments to improve their properties. The most promising strategies seem to be those in which it is made a partial replacement of the fossil materials, introducing small amounts of biocoke in the production of the anodes.^8,9^

The addition of biomass in the anodes helps to reduce the emissions of greenhouse gases and minimize the dependency on fossil materials as carbon sources.^8,10^ The increase of the biomass content, in the form of biopitch or biocoke (charcoal), tends to reduce the carbon anode properties. The replacement is made with low percentages and there is still a lot of research to be done until this alternative becomes industrially feasible.^4^

Binchotan charcoal is produced from a high-density type of Japanese oak and through a specific process of carbonization. Because of this, the material shows high density, good electrical properties, and a high fixed carbon content, in addition to low production of ashes and a good adsorption capacity, being typically used for cooking or as activated charcoal.^11,12^

This paper aimed to analyze the properties of carbon anodes made on a laboratory scale with the addition of binchotan charcoal to evaluate the replacement of small percentages of calcined petroleum coke.

## 2 Materials and Methods

The bioanode production and the analyses of the samples were performed based on the recommendations of the company R&D Carbon for the evaluation of Soderberg paste for aluminum application.^13^ The R&D Carbon is one of the biggest suppliers of solutions and an international reference in the field of carbon anodes. Providing support, supplies, and equipment for different types of analyses of carbon material that are used in the aluminum industry, following the appropriated international standards.^14^

### 2.1 Materials

Carbon anodes were produced on a laboratory scale with the addition of binchotan charcoal. Two samples of binchotan charcoal from different producers were used, one made in Japan and another in Myanmar. Both the charcoals were made from Ubame oak (*Quercus phillyraeoides*). The calcined petroleum coke and the coal tar pitch, used as raw material for carbon anodes, were provided by an aluminum company in Brazil. In this work, the anodes produced with the addition of binchotan charcoal are identified by the abbreviation AM, with charcoal made in Myanmar, and AJ, with charcoal made in Japan. The term bioanode, when used in this work, refers to a carbon anode produced with the addition of charcoal.

At first, a dry aggregate (blend) with calcined petroleum coke and binchotan was made, using binchotan proportions of 1 and 3%. The addition of binchotan was made in the finer fraction of the dry aggregate, in which the predominant granulometry is <200 mesh (<74 μm). This blend was mixed with coal tar pitch to form the Soderberg paste, also called green paste, in a proportion of 66.5% (blend) and 33.5% (pitch). The coke and pitch contents of the mixture were the same as those used by the company at the time and a total mass of 7 kg of green paste was produced with each type of binchotan.

To produce the carbon anodes, this paste was put into a metallic mold with a cylindrical shape and baked in the RDC-165 furnace from R&D Carbon. A given pressure was applied over the paste to guarantee a density level similar to the real process, and the temperature was gradually increased following different heating rates, until reaching 1000 °C. This process is known as anode calcination and can take up to 3 days, considering the cooling time. According to R&D Carbon,^13^ the laboratory anodes produced under these conditions show chemical and physical properties that are similar to those of industrial carbon anodes.

Subsequently, test specimens were produced for characterization, extracting cylinders of 50 mm diameter from the center of the anodes, to eliminate the ends and the outer layers that could have suffered reactions during the calcination due to the contact with the air and the walls of the mold. The lengths of the samples varied from 50 to 130 mm, depending on the analysis. The samples were produced in duplicate.

### 2.2 Characterizations

The characterizations were: apparent density, mechanical strength (flexural and compressive), CO_2_ reactivity, electrical resistivity, and chemical composition. The R&D Carbon apparatus that was used is identified by the company code (RDC-XXX), along with the corresponding standard.

#### 2.2.1 Apparent Density

The apparent density was calculated by the ratio of mass to volume, based on ISO 12985-1 (baked anode) and ISO 12985-2 (green paste). The green paste density was obtained using an Archimedes balance; mass was determined by weighing and volume by the Archimedes principle, by immerging the sample in water and measuring the resulting force. To obtain the baked anode density, a scale for weighing and an analog caliper for measuring the samples with Ø 50 × 130 mm were used.

#### 2.2.2 Mechanical Strength

To determine the mechanical strength, compressive strength, and flexural strength tests were performed. The analyses were made based on the standards ISO 18515 (compressive strength) and ISO 12986-1 (flexural strength). To obtain the compressive strength, the RDC-144 apparatus was used, and a load was applied until the breaking point of a sample with Ø 50 × 50 mm. The flexural test was performed using the RDC-187 apparatus and a sample measuring Ø 50 × 130 mm. The analysis was performed by the three-point method, where a load was applied in the center of the sample until the failure.

#### 2.2.3 CO_2_ Reactivity

The CO_2_ reactivity was analyzed with the RDC-146 furnace, where a sample with Ø 50 × 60 mm was exposed to a saturated atmosphere of carbon dioxide (CO_2_) for 7 h and under a temperature of 960 °C. After cooling, the sample was weighed and tumbled using the RDC-181 apparatus to remove any loose particles. The test was performed based on ISO 12988-1 and the result was presented by the amount of residual sample.

#### 2.2.4 Electrical Resistivity

The electrical resistivity of the anode was determined based on ISO 11713 and using the RDC-150 apparatus. A sample measuring Ø 50 × 130 mm was placed between two plates, a constant electrical current of 1 A was applied, and the voltage drop was measured. As the sample dimensions are known, the electrical resistivity can be obtained by calculations.

#### 2.2.5 Chemical Composition

The chemical composition was obtained with X-ray fluorescence, based on the NBR 15964,^15^ and using the equipment Axios Minerals of Panalytical. The contents of sodium (Na), silicon (Si), calcium (Ca), vanadium (V), iron (Fe), copper (Cu), sulfur (S), nickel (Ni), magnesium (Mg), and phosphorus (P) were obtained.

## 3 Results and Discussion

The full characterization of the binchotan charcoal, from Myanmar and Japan, was previously made and can be found elsewhere.^16^ For reference, some of the properties of these samples are presented in Table 1, such as carbon content, vibrated bulk density (VDB), and electrical resistivity. Both binchotan charcoal presented low electrical resistivity and high carbon content. For charcoal samples, the density was also considered high, with a higher value in the sample of Japan.

**Table 1:** Properties of the Binchotan Charcoal

	carbon content [%]	vibrated bulk density [g·mL^–1^]	electrical resistivity [μΩ·m]
binchotan (Myanmar)	87.2	0.555	1248
binchotan (Japan)	88.2	0.733	1350

The results of bioanode characterizations were compared with a typical industrial carbon anode, produced without charcoal and with the same pitch content (66.5%), and with a reference standard, data from the aluminum producer based on R&D Carbon company.^17^ A range of values that was acceptable for carbon anodes produced with calcined petroleum coke was indicated based on the reference.

It is worth mentioning that samples produced on a laboratory scale tend to present better results due to better control and efficiency of the mixture. The results of the many properties analyzed are listed in Table 2, for the bioanodes and the typical anode, along with the reference values.

**Table 2:** Properties of the Anodes

	bioanode				typical	reference
	AM 1%	AM 3%	AJ 1%	AJ 3%	carbon anode	R&D carbon
green density [g·mL^–1^]	1.575	1.563	1.576	1.568	1.575	1.520–1.590
baked density [g·mL^–1]^	1.440	1.460	1.480	1.480	1.450	1.390–1.470
compressive strength [MPa]	29.91	29.13	30.11	30.21	29.91	19.6−34.3
flexural strength [MPa]	7.52	8.93	8.78	9.03	9.1	6–10
CO_2_ reactivity [%]	93.7	87	93.9	90.1	93	80–92
electrical resistivity [μΩ·m]	64.5	67	66.2	61.5	66.4	68–76
electrical conductivity [kS·m^–1^]	15.50	14.93	15.11	16.26	15.06	13.16–14.71

A summary of the results of the properties analyzed in the anodes is in Figure 1, comparing the different contents of binchotan charcoal with the typical anode and the recommended range of values, indicated by the gray region.

**Figure 1:**
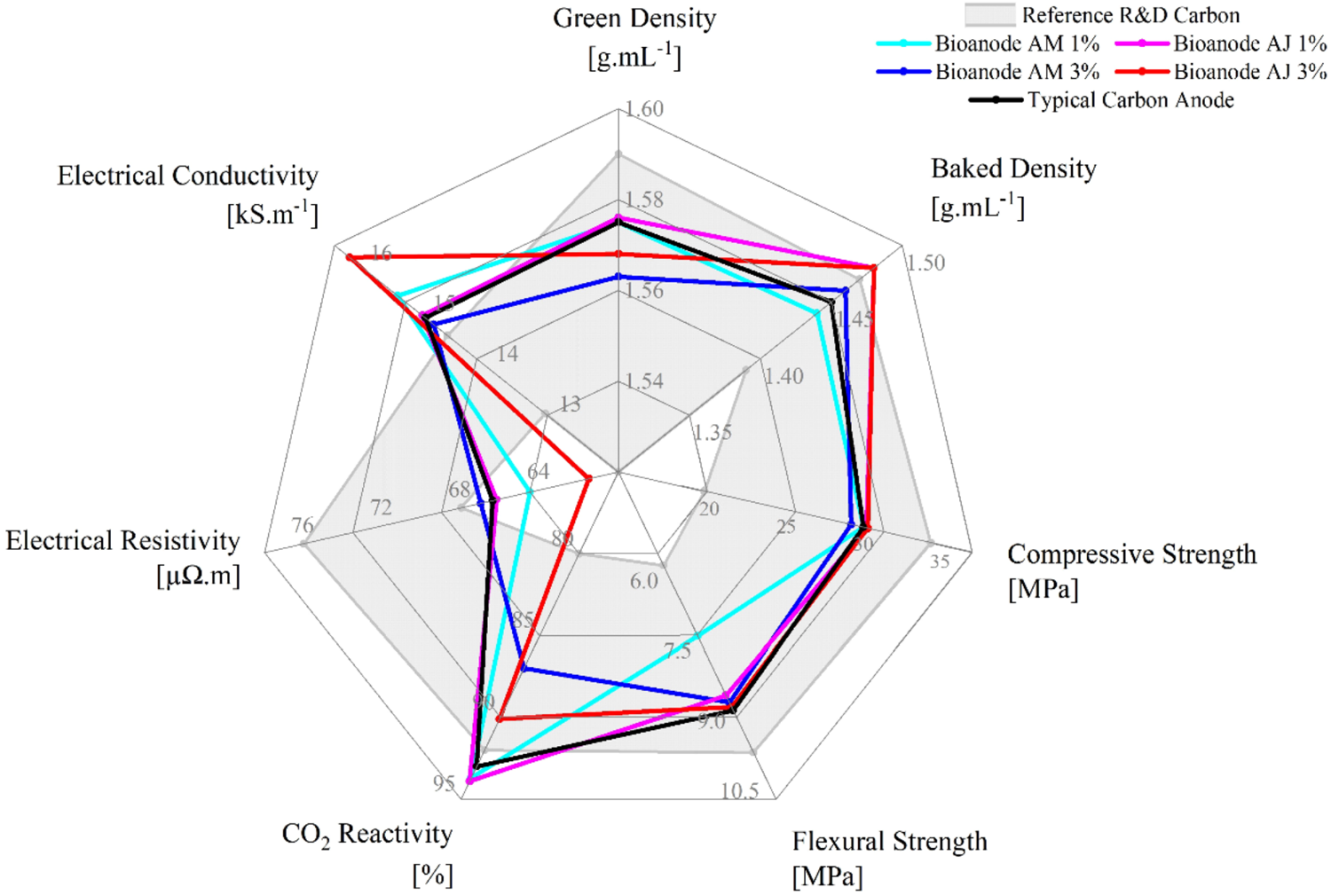
Comparison of the properties of the anodes.

### 3.1 Apparent Density

The green and baked densities of the bioanodes showed adequate values in comparison with the reference (Table 2). The baked density of the bioanode AJ was slightly above the typical anode and the recommended range, but it can be considered acceptable. Regarding the samples and the contents, the green density of the bioanodes with 1% was slightly higher than with 3%. There was no difference between the use of the binchotan from Myanmar and Japan. In the baked density, a bigger difference was observed between the samples, with a slightly higher value for the bioanode made with binchotan from Japan.

The addition of binchotan charcoal was made in the finer fraction of the dry aggregate. The use of a coarser granulometry can have an influence on the density and the other properties. It is necessary to re-evaluate the density if the parameters change. A high density is desirable because it contributes to the reduction of resistivity and prolongs the anode life, increasing the energy efficiency and carbon availability during the aluminum electrolysis.^9^ On the other hand, a too-high density can cause breakage in the anode due to the release of volatile matter during the baking process.^18^

### 3.2 Mechanical Strength

The results of the mechanical strength analysis, obtained by compressive and flexural tests, are listed in Table 2. These analyses are important to evaluate the mechanical behavior and the thermal shock resistance of the carbon anodes. Low mechanical strength, as well as the presence of cracks, can be detrimental to the performance of the anodes and even lead to their breakage. Very high values are indicative of brittle materials that are more sensitive to temperature variation.^14^

All the results obtained in the mechanical strength analysis were adequate and within the recommended range and close to those of the typical anode, especially for the compressive strength. The compressive strength values of the bioanodes were close; only the AM 3% sample showed a value slightly lower than the others. Regarding the flexural strength, the samples with 1% binchotan showed values a little lower than those with 3%, and the bioanodes AJ reached slightly bigger results.

The mechanical behavior in carbon anodes with charcoal observed in the studies of Hussein et al.^19^ and Huang et al.^20^ demonstrated that the increase of the charcoal content leads to a decrease in the strength, but with contents up to 3%, the changes were lower and acceptable. The results showed that the addition of up to 3% of binchotan charcoal did not cause negative impacts on the mechanical resistance of the anodes produced on a laboratory scale.

### 3.3 CO_2_ Reactivity

The results of the CO_2_ reactivity tests are listed in Table 2. The results were indicated in percentage and refer to the amount of material that remained after the analysis. Thus, a lower value indicates a higher reactivity, resulting in greater consumption of the anode.

All the bioanodes showed satisfactory results, indicating that the wear caused by the CO_2_ reactivity was not impaired by the addition of the binchotan charcoal. The typical anode and the samples with 1% binchotan showed a lower reactivity, with values slightly above the reference itself. In general, charcoal is more reactive than petroleum coke.^20–22^ The increase in the charcoal content tends to increase the CO_2_ reactivity. This tendency was observed in the bioanodes with 3% of binchotan.

A high reactivity can lead to higher net anode consumption, increasing the electrical resistivity during the electrolysis, reducing the energy efficiency of the process, and increasing the CO_2_ emissions of aluminum production.

As well as charcoal, coal tar pitch is usually more reactive than calcined petroleum coke. In this case, a high reactivity results in greater consumption of the binder matrix, causing the phenomenon known as dusting. This phenomenon is characterized by the detachment of coke particles due to the loss of structural integrity of the anode.^20^ The occurrence of dusting causes an increase in the electrical resistivity of the electrolysis process. According to Ozturk et al.,^5^ the theoretical amount of carbon needed to produce 1 kg of aluminum is about 0.33 kg. However, other reactions of carbon with air and CO_2_ occur, and more than 0.40 kg of carbon is consumed for every 1 kg of aluminum produced. Because of this, anode reactivity is related to excess anode consumption and higher greenhouse gas emissions.

### 3.4 Electrical Resistivity

The properties of the carbon anodes are strongly affected by the properties of the coke. In such a way that the electrical resistivity of the anode is associated with that of coke and, by extension, with its density too. These parameters are indicative of high-quality materials, and anodes that show proper values of density and electrical resistivity contribute to enhancing the energy efficiency of the process.^9^ The results of electrical resistivity and conductivity are in Table 2. The conductivity was indicated to help the comparison with other studies because it is the reciprocal of the resistivity.

As with the typical anode, the electrical resistivities of the bioanodes were below the reference values; consequently, the electrical conductivities were also higher than the reference. However, the results were considered acceptable because they indicate that the samples have good electrical properties. According to Amara et al.,^21^ a low value of resistivity is wanted during the aluminum electrolysis process due to its influence on energy consumption and production cost.

The bioanode AM 1% showed an electrical resistivity lower than the bioanode AM 3%. For the sample AJ, the lower value of resistivity was obtained with 3% of binchotan (61.5 μΩ·m), which was also lower than the others. It is important to highlight that fine particles of charcoal were used to replace the calcined petroleum coke. If the grain size or the content of charcoal were changed, the standard recipe of the anode needs to be re-evaluated to adjust the pitch content employed, which could lead to a change in the mechanical and electrical properties.^19^

The bioanode properties are affected by the charcoal properties. Given the high values of density noticed in the bioanodes and the fact that binchotan is a kind of charcoal with high density and good electrical conductivity, the results of the electrical properties tests were expected to be adequate, as was seen in this analysis.

### 3.5 Chemical Composition

The results of the chemical composition are listed in Table 3, along with the reference values for comparison.

**Table 3:** Chemical Composition of the Anodes

	bioanode				typical	reference
	AM 1%	AM 3%	AJ 1%	AJ 3%	carbon anode	R&D carbon
Na [ppm]	43	39	94	56	46	50–150
Si [ppm]	277	260	176	154	159	100–300
Ca [ppm]	82	78	185	117	56.5	50–150
V [ppm]	169	167	183	169	148	40–240
Fe [ppm]	247	244	194	188	204	100–500
Cu [ppm]	3	3	3	3	3	
S [%]	0.43	0.42	0.42	0.40	0.74	0.5–1.5
Ni [ppm]	134	135	138	130	118	80–160
Mg [ppm]	9	8	6	4	0	
P [ppm]	1	1	1	8	2	0–10

Most of the values were within the recommended range. For some elements, the difference was not significant, as in the sodium (Na) content of the bioanodes with 1% of charcoal (<50 ppm), the content of calcium (Ca) of bioanode AM 3% (185 ppm), and in the sulfur (S) contents of all binchotan samples (≈0.42%). A reference value for copper (Cu) and magnesium (Mg) was not found, however, the results were very low (<10 ppm).

A comparison of the main elements analyzed is shown in Figure 2, making it evident that the bioanodes presented mostly satisfactory results. Copper and magnesium were not included due to the absence of a reference value. The recommended range is indicated by the gray region.

**Figure 2:**
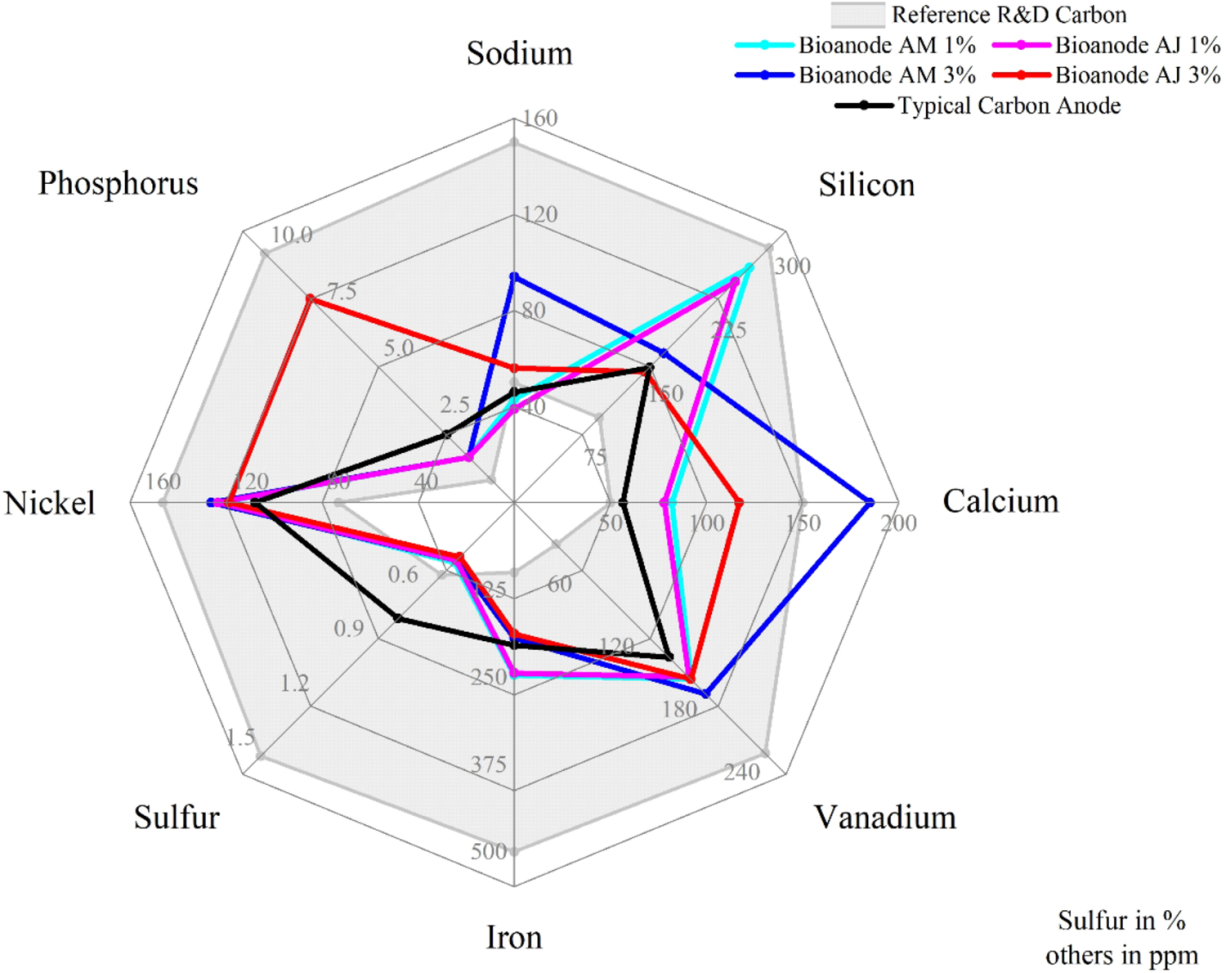
Comparison of the main elements of the anodes.

No tendency was noted in the results given the increase in the charcoal content, from 1 to 3%. Some elements showed higher values with the increase of the charcoal content, others showed a reduction, and there were also those in which the variation was zero or practically null.

Regarding the chemical composition, the bioanodes produced with the addition of binchotan charcoal showed acceptable values on a laboratory-scale production. For large-scale experiments, it is necessary to track the elements that exhibited deviation from the recommended range, as well as the elements that typically have a higher concentration in charcoal, such as sodium, silicon, calcium, and phosphorus.

### 3.6 Industry Implications

As previously discussed, the values that deviated from the reference did not compromise the results, such as the higher electrical conductivity of all bioanodes and the higher values of baked density and CO_2_ reactivity of some samples.

The laboratory tests provided valid information to analyze the characteristics of carbon anodes produced with the addition of binchotan charcoal. However, large-scale tests may present different values, given the possibility of a greater variation in the parameters due to the use of larger equipment and a greater variation in the characteristics of the feedstocks. In a laboratory, the results tend to be better because it is possible to have better control over the process and a higher efficiency in the mixture of materials. More tests are needed, with different scales and different contents of charcoal, to validate the use of binchotan charcoal as a partial substitute for the calcined petroleum coke in the aluminum industry on a large scale.

Another advantage of the use of binchotan is the fact that a commercial sample can be used naturally, i.e., the material does not need any modification or treatment. Only the mechanical processing used to adjust the granulometry was required to formulate the green paste.

It is important to note that the content of the coal tar pitch used to form the anode paste influences the final quality of the carbon anode. Hussein et al.^19^ and Hussein et al.^23^ verified that the increase in pitch content in the formulation of the anode with the use of biomass led to an increase in density and mechanical strength, as well as a decrease in electrical resistivity. This could be attributed to a better bonding of the coke particles due to a greater availability of the binder.

There is an amount of pitch that is considered ideal. The pitch demand must be enough to bond the coke particles, fill free spaces, and allow the pitch expansion during the baking, preventing excessive shrinkage or expansion and the formation of cracks or porosity in the structure of the carbon anode. A low pitch content hinders the formation of the anode paste, resulting in anodes with low density and lack of homogeneity. High-pitch contents led to a drop in the baked density due to a greater loss of mass during the baking, which increases the porosity.^9^ Modifications in the pitch contents are mainly required when there is a decline in the quality of the raw materials. Just as can happen with petroleum coke, the commercial samples of binchotan charcoal are also susceptible to quality variability.

The use of biomass in the production of carbon anodes is one of the alternatives being studied to try to reduce CO_2_ emissions from aluminum production. Another alternative that is being implemented and has great potential is the use of inert anodes as a substitute for prebaked anodes. The inert anode is not carbon-based, so the CO_2_ is not released from the reaction during aluminum electrolysis. Two projects have gained attention in this type of technology: the Elysis project, developed by a partnership between the companies Alcoa and Rio Tinto and expected to be commercialized in the next years; and the company US Rusal, which is currently testing on an industrial scale.^1,9^

The replacement by inert anodes is not simple in companies that employ the Soderberg technology of continuous anodes; it is necessary to modify the infrastructure and change equipment. In this case, the addition of biomass to carbon anodes becomes an even more appealing alternative, even if it is done in small percentages.

Considering the large volume of petroleum coke that is used in the aluminum industry, even the addition of just 1% of charcoal can be beneficial to this field. According to Edwards et al.,^24^ the best electrolytic cells in the world can achieve a net carbon consumption of approximately 395 kg per ton of aluminum, but in reality, this value is typically between 400–450 kg. This carbon comes from the mixture of coke and pitch used in the anode and, based on a generic recipe with 70% of coke and 30% of pitch, it can be estimated that an average of 298 kg of calcined petroleum coke is used per ton of primary aluminum. For a replacement from 1 to 3% of charcoal, would be needed between 2.98 and 8.94 kg of charcoal for every 1 ton of aluminum produced. In 2021, 345 thousand tonnes of primary aluminum were produced in Brazil using Soderberg technology.^25^ Considering this volume, about 1028 to 3084 tonnes of charcoal would be necessary for a substitution of 1 to 3% of coke.

The real impact that the addition of charcoal would have on carbon emissions needs to be analyzed through a life cycle assessment. The carbon demand in the aluminum industry is high, so an analysis of the feasibility of using binchotan charcoal on a large scale must also consider the costs involved, the logistics, and its production capacity. This paper focused on the properties of binchotan to investigate its technical feasibility. Binchotan is known for being expensive, and for an industrial-scale application would be necessary white charcoal with high availability and a competitive price. One possibility could be to try to replicate the production process of binchotan using local hardwood.

From a technical point of view, the results indicated that binchotan charcoal had the necessary characteristics to be used as a substitute, in small percentages, for calcined petroleum coke in the production of carbon anodes on a laboratory scale. Based on this data, new studies can be carried out to find other sources of biomass that can be used and that meet the demands of the aluminum industry. One of the determining factors in this case is the proximity of the charcoal production site to the aluminum production plant. A large-scale analysis could also provide information about the performance and the lifetime of the bioanodes.

## 4 Conclusions

The physicochemical characteristics presented by the carbon anodes produced with the addition of binchotan charcoal were close to the typical anode and within an acceptable range of the reference values. Given the great volume of primary aluminum production, even the use of only 1% of charcoal in the production of the anodes could have a big impact on the emissions of greenhouse gases. Also, it is a starting point in the study of materials that can reduce the dependence on nonrenewable sources. These results evidenced that, from a technical point of view, the addition of small percentages of binchotan charcoal seemed to be feasible. However, more studies to evaluate the economic and logistical feasibility of using binchotan charcoal on a large scale are still necessary.

## References

[1] Brough D., Jouhara H. (2020). The aluminium industry:
A review on state-of-the-art
technologies, environmental impacts and possibilities for waste heat
recovery. Int. J. Thermofluids.

[2] Venditti, N. L. B. Visualizing the Abundance of Elements in the Earth’s Crust. Elements.. https://elements.visualcapitalist.com/elements-in-the-earths-crust-abundance/ (accessed Sep 9, 2024).

[3] Kvande H., Drabløs P. A. (2014). The Aluminum
Smelting Process and Innovative Alternative
Technologies. J. Occup. Environ. Med..

[4] Saevarsdottir G., Kvande H., Welch B. J. (2020). Aluminum
Production in the Times
of Climate Change: The Global Challenge to Reduce the Carbon Footprint
and Prevent Carbon Leakage. JOM.

[5] Ozturk S., Kocaefe D., Bhattacharyay D., Kocaefe Y., Morais B. (2018). Modification
of coke by different additives to improve anode properties. Fuel.

[6] Glastonbury R. I., Beukes J. P., van Zyl P. G., Tangstad M., Ringdalen E., Dall D., Steenkamp J. D., Mushwana M. (2021). Characterisation of
a Real-World Søderberg Electrode. Metals.

[7] Petrobras . Coque Verde de Petróleo - Informações Técnicas; Petrobras, 2019; pp 1–9.

[8] Senanu S., Solheim A. (2021). Biocarbon in the Aluminium Industry: A Review. Miner., Met., Mater., Ser..

[9] Ratvik A. P., Mollaabbasi R., Alamdari H. (2022). Aluminium production process: from
Hall–Héroult to modern smelters. ChemTexts.

[10] Adrados A., De Marco I., López-Urionabarrenechea A., Solar J., Caballero B., Gastelu N. (2016). Biomass Pyrolysis Solids
as Reducing Agents: Comparison with Commercial Reducing Agents. Materials.

[11] Fujimoto, N. Binchotan Charcoal: How it’s made and why it’s awesome. Knifewear. https://knifewear.com/blogs/articles/binchotan-charcoal-how-it-is-made-and-why-it-is-awesome (accessed Aug 12, 2024).

[12] Itani S., Kishimoto N. (2018). Preparation
of Ubamegashi Activated Carbon by Using
Superheated Steam Carbonization/Activation Processes. J. Chem. Eng. Jpn..

[13] R&D Carbon . Evaluation of Söderberg Paste for Aluminum Application: Samples Preparation and Test Methods. 2012, pp 1–16.

[14] R&D Carbon . Catalogue of Equipment and Standards: Our Test Equipment Used in the Carbon Electrode Industry. 2023, pp 1–70.

[15] NBR 15964 . Minérios de alumínio — Determinação da composição química — Método da espectrometria de fluorescência de raios X por comprimento de onda. 2011.

[16] Pereira L. C., Corrêa C. R., Zilnyk K. D., Hias E. O., Santos H. C., Yamamoto H., Barros J. L., Yamaji F. M. (2024). Binchotan Charcoal
as an Alternative to Calcined Petroleum Coke in Anodes in the Aluminum
Industry. ACS Sustainable Chem. Eng..

[17] R&D Carbon . Anodes for the Aluminum Industry 1995–2005, 2nd ed.; R&D Carbon Ltd.: Sierre, Switzerland, 2006.

[18] Amara B., Kocaefe D., Kocaefe Y., Bhattacharyay D., Côté J., Gilbert A. (2022). Effect of Coke Type on Partial Replacement
of Coke with Modified Biocoke in Anodes Used in Primary Aluminum Production. Light Metals.

[19] Hussein A., Fafard M., Ziegler D., Alamdari H. (2017). Effects of Charcoal
Addition on the Properties of Carbon Anodes. Metals.

[20] Huang X., Kocaefe D., Kocaefe Y. (2018). Utilization of Biocoke as a Raw Material
for Carbon Anode Production. Energy Fuels.

[21] Amara B., Faouzi F., Kocaefe D., Kocaefe Y., Bhattacharyay D., Côté J., Gilbert A. (2021). Modification of biocoke destined
for the fabrication of anodes used in primary aluminum production. Fuel.

[22] Monsen, B. E. ; Ratvik, A. P. ; Lossius, L. P. Charcoal in anodes for aluminium production TMS Light Metals 2010, pp 929–934.

[23] Hussein A., Picard D., Alamdari H. (2021). Biopitch as
a Binder for Carbon Anodes:
Impact on Carbon Anode Properties. ACS Sustainable
Chem. Eng..

[24] Edwards L., Hunt M., Weyell P., Nord J., Côté J., Coulombe P., Morais N. (2022). Quantifying the Carbon
Footprint
of the Alouette Primary Aluminum Smelter. JOM.

[25] ABAL . Estatísticas Nacionais: Alumínio Primário. ABAL. http://abal.org.br/estatisticas/nacionais/aluminio-primario/ (accessed Apr 28, 2024).

